# A hypercoagulable state leading to venous limb gangrene associated with occult lung adenocarcinoma

**DOI:** 10.1002/ccr3.2106

**Published:** 2019-03-25

**Authors:** Matthew M. A. Waite, Anthony W. Martinelli, Stephen D. Preston, Emma Gudgin, Emily Symington, Robert C. Rintoul, Adam Peryt, Patrick Coughlin, Paul Hayes, David Gilligan, Martin Besser

**Affiliations:** ^1^ Addenbrooke's Hospital University of Cambridge School of Clinical Medicine Cambridge UK; ^2^ Department of Thoracic Oncology Papworth Hospital NHS Foundation Trust Cambridge UK; ^3^ Department of Haematology Papworth Hospital NHS Foundation Trust Cambridge UK; ^4^ Department of Thoracic Surgery Papworth Hospital NHS Foundation Trust Cambridge UK; ^5^ Department of Vascular and Endovascular Surgery Addenbrooke's Hospital Cambridge UK

**Keywords:** argatroban, dabigatran, hematology, hypercoagulability, lung adenocarcinoma, respiratory

## Abstract

We report a case of lung adenocarcinoma‐associated hypercoagulability leading to venous limb gangrene, managed successfully with argatroban and then dabigatran. Use of idarucizumab permitted diagnostic investigations, leading to targeted antineoplastic therapy with crizotinib, surgical resection with curative intent, and continued survival over 2 years after the index event.

## 1 INTRODUCTION

A 31‐year‐old woman presented to the Emergency Department with a painful, cold, blue left leg with absent distal pulses. Four days previously, she had been diagnosed with a deep vein thrombosis (DVT) by Doppler ultrasound after presenting with left leg swelling and had been commenced simultaneously on low‐molecular‐weight heparin (LMWH, Dalteparin 200 U/kg) and warfarin loading (10, 5, and 5 mg). Aside from a 3‐month history of recurrent thrombophlebitis, she was formerly fit and well with no significant past medical or family history and had never smoked. She had had two successful pregnancies and one miscarriage 10 years previously. Her INR was 5.78 with a platelet count of 205.

**Figure 1:**
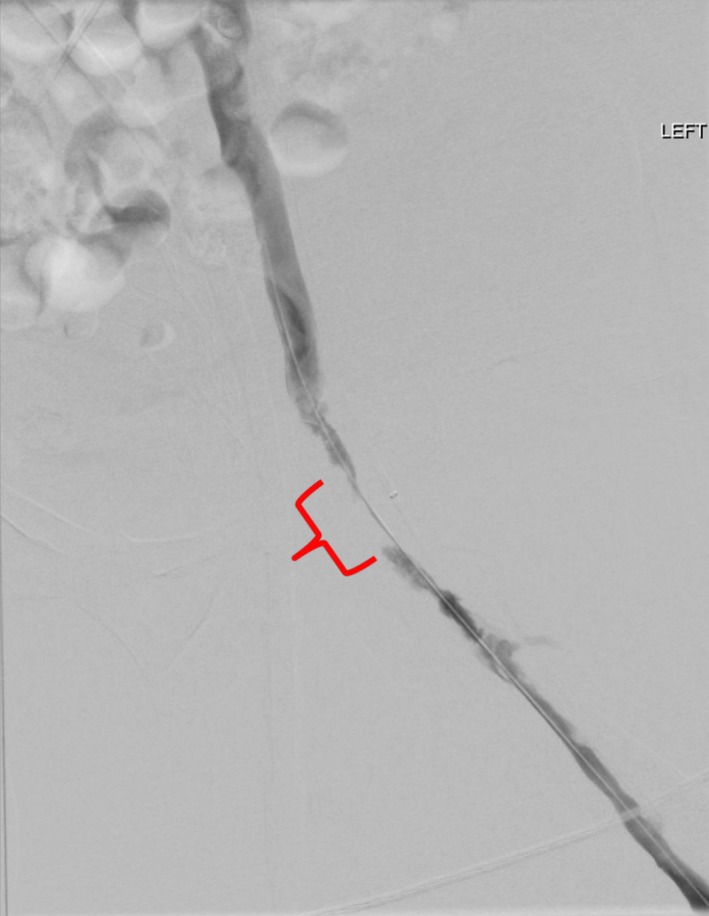
Angiogram showing reduced flow in the left common femoral vein due to thrombus (indicated)

A CT angiogram confirmed an extensive left leg DVT and secondary arterial compromise leading to venous gangrene (Figure 1). There was preserved flow in the popliteal artery, but no detectable arterial flow in the foot, and there were thromboses in posterior tibial and popliteal veins. Although directed thrombolysis with alteplase and heparin provided some benefit, the limb was not viable and the patient underwent an above knee amputation the following day. A heparin infusion was commenced and maintained to an aPTT of 45‐60 seconds for 72 hours while gradual recovery from the high INR was awaited. LMWH and warfarin were recommenced 96 hours after presentation when the patient's INR was <2.0. Thrombocytosis ensued (to a peak of 710 × 10*9/L), and the INR rose to 5.4 in the next 72 hours.

She was investigated for an underlying malignancy (breast exam, abdominal ultrasound, and tumor markers). A thrombophilia screen identified a heterozygous factor V Leiden mutation and positive anticardiolipin antibodies (ACA IgG 48 GPLU/mL, >99th centile). Low levels of Proteins C and S were assumed to be due to warfarin therapy. An Acustar^®^ chemoluminescence assay for HIT antibodies was negative.

**Figure 2:**
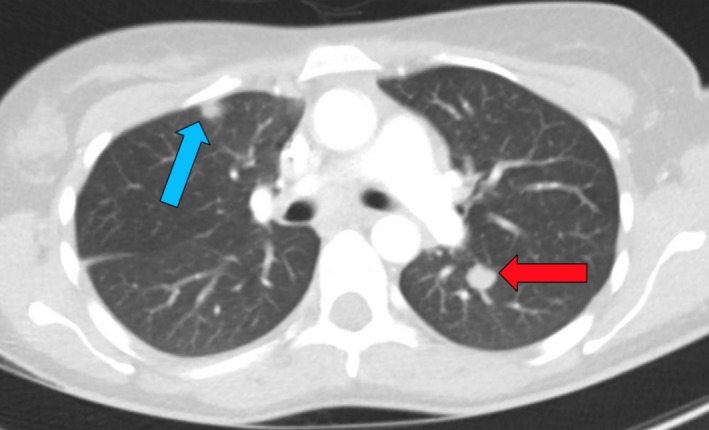
CT scan of chest identifying a pulmonary lesion (red) and an infarct (blue) due to a pulmonary embolus

The patient was diagnosed with further DVTs in her right arm and right internal jugular vein, and a CT pulmonary angiogram, performed due to persistent tachycardia, revealed pulmonary emboli. The patient was switched to fondaparinux 7.5 mg and underwent plasmapheresis for a potential diagnosis of catastrophic antiphospholipid antibody syndrome (CAPS). The final report of her CT pulmonary angiogram identified an 11 mm left lower lobe lung lesion with mediastinal and hilar lymphadenopathy (Figure 2).

Despite a therapeutic anti‐Xa level on fondaparinux (1.03 IU/mL, measured 4‐5 hours postinjection), the patient developed a further thrombosis in her right common femoral vein. A decision was made to switch her onto an argatroban infusion. Argatroban is a direct thrombin inhibitor, licensed for HIT, with a half‐life of 45 minutes and monitored by the aPTT, with no specific reversal agent. Despite being irreversible, the short half‐life allowed CT‐guided needle biopsy of the previously identified left lower lobe lung lesion. Argatroban was discontinued 6 hours preprocedure and restarted 8 hours postprocedure. While there was no bleeding complication, the patient developed a symptomatic left superior caval vein thrombosis, associated with a peripherally inserted central catheter. Histopathological analysis of the lesion showed a moderate to poorly differentiated adenocarcinoma with strong diffuse staining for thyroid transcription factor 1 (TTF1) and cytokeratin 7 (CK7), consistent with an adenocarcinoma of lung origin.

**Figure 3:**
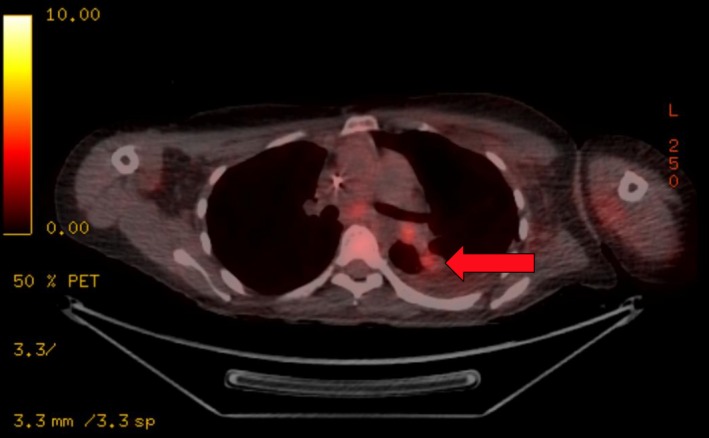
A PET‐CT scan showing FDG‐uptake into the pulmonary lesion (arrow), with associated lymphadenopathy

Staging with PET‐CT (Figure 3) showed ^18^FDG uptake in the left lower lobe lesion as well as in mediastinal and ipsilateral hilar lymph nodes (stations 4L, 6, 7, 10L, and 11L). If this nodal ^18^FDG‐avidity was confirmed as due to spread of cancer, this would have precluded radical surgical treatment. Therefore, the argatroban infusion was again interrupted to allow for endobronchial ultrasound‐guided transbronchial needle aspiration (EBUS‐TBNA) of the station 4L and seven nodes. These biopsies confirmed T1a N2 M0 adenocarcinoma of lung origin (TTF1 positive with an ALK translocation).

The patient was converted to dabigatran 150 mg BD, as it was felt to resemble most closely the action of argatroban, although the failure of LMWH anticoagulation may have been related to the warfarin‐induced drop in Protein C. Oncological review determined that the patient should receive chemotherapy with crizotinib (a tyrosine kinase inhibitor), due to the presence of an EML4‐ALK translocation of the adenocarcinoma, despite her WHO performance status of three (attributable to the recent amputation and ongoing inpatient stay). There was concern regarding a potential interaction between crizotinib and dabigatran as the former has been shown to act as an inhibitor of P‐glycoprotein and the latter is a P‐glycoprotein substrate.^1^, ^2^ The theoretical risk of increased dabigatran levels was not observed: levels were measured by calibrated diluted thrombin assay (Hyphen^®^) on multiple occasions 90 minutes postadministration of dabigatran, but no detectable dabigatran was demonstrated. There were no clinical concerns over the subsequent 9 months of combination therapy.

Repeat imaging after her second cycle of crizotinib showed significant response in terms of tumor size, as well as resolution of her thromboses. After six cycles of crizotinib, PET‐CT indicated an excellent metabolic response with a reduction in the primary lesion's size to 8 × 6 mm (with no significant tracer uptake) and resolution of the mediastinal and hilar lymph node enlargement. After seven cycles, EUS‐TBNA/EUS(B)‐FNA showed that the only lymph node amenable to biopsy was at mediastinal station 7 (subcarinal), which contained no malignancy. After eight cycles, mediastinoscopy was also negative for malignancy and so, after nine cycles, she proceeded to a uniportal VATS left lower lobectomy and lymphadenectomy. The decision to proceed to surgery was in view of the clinical complete response to crizotinib and drug‐related toxicity of CTCAE grade 2 dysgeusia.

Dabigatran was successfully bridged with idarucizumab with no concern of external bleeding for her restaging biopsies, with treatment restarted 8 hours postprocedure at 150 mg. For the lobectomy, the patient was again bridged with idarucizumab, but postoperatively treated with argatroban for 24 hours prior to recommencing dabigatran.

**Figure 4:**
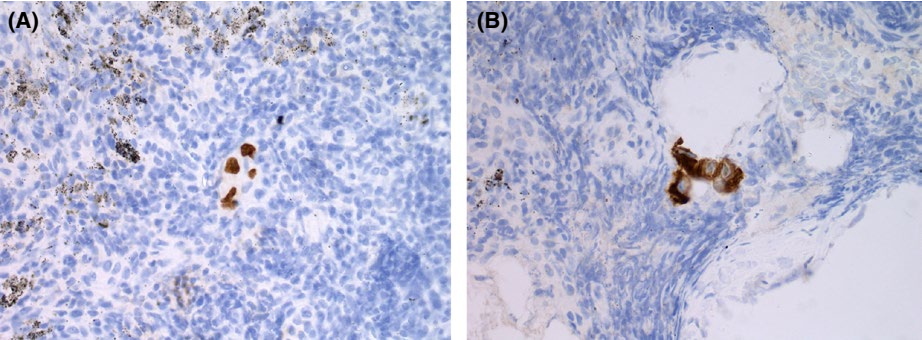
Postoperative histology from station five lymph nodes, using brown immunohistochemistry showing isolated tumor cells. A, Thyroid transcription factor 1 (TTF1) nuclear staining. B, Cytokeratin (MNF116) cytoplasmic staining

Postoperative pathology revealed areas of fibrosis but no active tumor in the lung and isolated single cell deposits in station 5 lymph nodes [Figure 4], staged as ypT0 ypN0 (i+). It was not possible to test these cells for ALK translocation by IHC or FISH. A shared decision was made to commence standard adjuvant chemotherapy, and vinorelbine/cisplatin was commenced for two cycles, followed by a change to vinorelbine/carboplatin for the final two cycles due to drug toxicity.

At the time of writing, the patient remains under long‐term thoracic oncology follow‐up with her most recent imaging, over 2 years after initial presentation, showing no evidence of cancer recurrence.

## 2 DISCUSSION

An unprovoked DVT in a young person should always raise the possibility of a thrombophilia or an undiagnosed cancer. Outpatient management of DVT is now the standard of care, and investigations for any underlying cause are fast‐tracked rather than performed on initial presentation. Warfarin and LMWH have an exceedingly low rate of failure,^3^ and representation within 96 hours of commencing anticoagulation is likely due to one of four differential diagnoses: cancer, antiphospholipid syndrome, heparin‐induced thrombocytopenia (HIT), or noncompliance. The 3‐month history of recurrent thrombophlebitis could also suggest Trousseau's syndrome (recurrent thrombophlebitis arising from the formation of small blood clots in association with malignancy). Her INR was raised, suggesting that she was not overtly hypercoagulable. The presentation with an acutely painful limb with preserved pulses could also alert the clinician to phlegmasia coerulea dolens: the classic presentation is with detectable pulses; however, venous and microvascular thrombosis will eventually reduce the runoff from the small distal arteries.

A heterozygous factor V Leiden mutation is unlikely to be a sufficient cause of this clinical presentation but may contribute to a prothrombotic state by making the hypercoagulable state more difficult to control, particularly in the context of the warfarin‐induced inactivation of vitamin K‐dependent clotting factors. Warfarin therapy interferes with vitamin K epoxide reductase and induces a rapid drop in all vitamin K‐dependent clotting proteins, affecting Protein C (half‐life 5 hours) before Factor VII (half‐life 7 hours) and the other clotting factors II, IX, and X (half‐lives 24‐60 hours). Antiphospholipid antibodies could promote platelet activation and exacerbate endothelial injury as part of a possible antiphospholipid syndrome (which may further be associated with the previous miscarriage). Although the acute symptomatic presentation is unusually severe and the patient did not have disseminated intravascular coagulation (Asherson's syndrome), the borderline positive result led to the patient being treated with plasmapheresis in the hope of stopping the prothrombotic state.

However, the patient had a thrombotic event postplasmapheresis and did not have the complete features of CAPS. Fondaparinux is not recommended in cancer‐associated thrombosis and has proved insufficiently effective in this case despite therapeutic Anti‐Xa levels.

Having failed two antithrombin‐dependent Xa inhibitors, argatroban (which directly inhibits thrombin) was chosen for its pharmacokinetic profile, where it reaches steady state over 1‐3 hours, and has a half‐life of approximately forty minutes. This allowed biopsy of her lesion and was the only drug that appeared to control her hypercoagulable state.

Dabigatran was chosen as it is pharmacologically similar to argatroban but can dose orally. Although further study is needed, the interaction between crizotinib and dabigatran may not be clinically relevant. A number of drug interactions listed in the DOAC Summary of Product Characteristics remain theoretical.

Venous gangrene is rare, but there is a significant association with malignant disease.^4^ In accord, it seems that there is a particular relationship between DVTs and ALK‐positive lung neoplasms,^5^ as seen in this patient. Ten patients have been described^6^ who suffered severe venous limb ischemia/venous leg gangrene after commencing warfarin treatment for a metastatic‐adenocarcinoma‐related DVT, and this patient may fit this presentation. Of note, these patients experienced the same thrombocytosis as our patient on starting the warfarin/heparin combination therapy. It is likely that the heterozygosity for factor V Leiden and IgG anticardiolipin antibodies, although each insufficient in isolation to cause this degree of hypercoagulability, will have contributed to the presentation.

Mechanisms of cancer‐related hypercoagulability appear diverse.^7^, ^8^ Currently, warfarin is the treatment of choice in patients with an unprovoked DVT. Commencing warfarin is complicated by the induction of a transient prothrombotic state through critical reduction in anticoagulants protein C and S prior to reduction in the clotting factors II, VII, IX, and X. LMWH remains the anticoagulant of choice in cancer‐associated DVT although edoxaban, an oral Xa inhibitor, was shown to be noninferior for cancer‐associated DVT.^9^ In our own practice, we underutilize dabigatran for VTE despite the availability of a specific reversal agent which proved to have high clinical utility in this case on three occasions including for lobectomy. It is possible that conversion to another DOAC would be an option after achieving remission from the lung cancer.

The similarities between this patient with an adenocarcinoma of the lung and the series described by Warkentin et al^6^ are intriguing. The warfarin failed to control hypercoagulability (despite the supratherapeutic INR) and likely contributed to venous limb gangrene by compromising the ability of the protein C anticoagulant system to downregulate thrombin in the microvasculature. The thrombocytosis observed may describe a rebound from the reduction in prothrombotic state when effective pharmacologic anticoagulation was achieved (upon resumption of LMWH), thus reducing platelet activation and consumption.

We describe for the first time the successful successive use of direct thrombin inhibition with argatroban followed by oral direct thrombin inhibition with dabigatran. The changing therapeutic landscape that has now given us access to flexible and rapidly reversible antithrombin independent thrombin inhibition has opened a new armamentarium of anticoagulation and bridging which we are yet to fully utilize. The absence of detectable dabigatran during the combined therapy with dabigatran and crizotinib highlights the ongoing issues with reliably measuring and interpreting dabigatran levels in the small group of patients that requires these. It also highlights that the patient's potential benefit from drug combination therapy sometimes justifies the risk of causing potential interaction provided mitigating interventions are put in place to detect potentially significant problems.

The efficacy of direct oral anticoagulants in the treatment of cancer‐associated thrombosis is currently being established. Two recent studies have investigated the utility of factor Xa inhibitors, namely rivaroxaban, in cancer‐associated venous thrombosis.^10^, ^11^ Both appear to conclude similar rates of recurrent thromboembolic events when comparing rivaroxaban to both heparin and warfarin. It may be that the oral anticoagulants will adopt the role of the older anticoagulants in cancer‐associated thrombosis in the future.

## CONFLICT OF INTEREST

None declared.

## AUTHOR CONTRIBUTION

MW: Primary manuscript author. AM: Involved in the writing from a thoracic oncology point of view. SP: Managed the patient and provided much of the histopathological information in the manuscript. EG: Managed the patient and involved in editing the manuscript. ES: Managed the patient and involved in editing the manuscript. RR: Managed the patient and involved in editing the manuscript. AP: Managed the patient and involved in editing the manuscript. PC: Managed the patient and involved in editing the manuscript. PH: Managed the patient and involved in editing the manuscript. DG: is a senior author from a thoracic oncology aspect and managed the patient. MB: is a overall senior author and managed the patient.
